# Interleukin-34 and immune checkpoint inhibitors: Unified weapons against cancer

**DOI:** 10.3389/fonc.2023.1099696

**Published:** 2023-01-31

**Authors:** Fadhl Alshaebi, Mohammed Safi, Yousif A. Algabri, Mahmoud Al-Azab, Abdullah Aldanakh, Mohammed Alradhi, Alariqi Reem, Caiqing Zhang

**Affiliations:** ^1^ Department of Respiratory, Shandong Second Provincial General Hospital, Shandong University, Jinan, Shandong, China; ^2^ Department of Biomedical Engineering, School of Control Science and Engineering, Shandong University, Jinan, Shandong, China; ^3^ Department of Immunology, Guangzhou Institute of Pediatrics, Guangzhou Women and Children’s Medical Center, Guangdong Provincial Clinical Research Center for Child Health, Guangzhou Medical University, Guangzhou, China; ^4^ Department of Urology, First Affiliated Hospital of Dalian Medical University, Dalian, Liaoning, China; ^5^ Department of Urology, The Affiliated Hospital of Qingdao Binhai University, Qingdao, Shandong, China; ^6^ Faculty of Medicine and Health Sciences, Amran University, Amran, Yemen

**Keywords:** immune checkpoint inhibitors (ICIs), interluikin-34 (IL-34), tumor microenvironment, programmed cell death protein 1 (PD-1), cytotoxic T-lymphocyte-associated protein 4 (CTLA-4), cancer therapy

## Abstract

Interleukin-34 (IL-34) is a cytokine that is involved in the regulation of immune cells, including macrophages, in the tumor microenvironment (TME). Macrophages are a type of immune cell that can be found in large numbers within the TME and have been shown to have a role in the suppression of immune responses in cancer. This mmune suppression can contribute to cancer development and tumors’ ability to evade the immune system. Immune checkpoint inhibitors (ICIs) are a type of cancer treatment that target proteins on immune cells that act as “checkpoints,” regulating the activity of the immune system. Examples of these proteins include programmed cell death protein 1 (PD-1) and cytotoxic T-lymphocyte-associated protein 4 (CTLA-4). ICIs work by blocking the activity of these proteins, allowing the immune system to mount a stronger response against cancer cells. The combination of IL-34 inhibition with ICIs has been proposed as a potential treatment option for cancer due to the role of IL-34 in the TME and its potential involvement in resistance to ICIs. Inhibiting the activity of IL-34 or targeting its signaling pathways may help to overcome resistance to ICIs and improve the effectiveness of these therapies. This review summarizes the current state of knowledge concerning the involvement of IL-34-mediated regulation of TME and the promotion of ICI resistance. Besides, this work may shed light on whether targeting IL-34 might be exploited as a potential treatment option for cancer patients in the future. However, further research is needed to fully understand the mechanisms underlying the role of IL-34 in TME and to determine the safety and efficacy of this approach in cancer patients.

## 1 Introduction

IL-34 is a human protein that was discovered in 2008 by Lin and his colleagues (1). It is a hypothetical protein that may be accessed in a public database (C16orf77) and was reported as a selective protein that binds CD14^+^ monocytes and enhances their survival (1–3). Several reports have shown that incubating IL-34 with the extracellular domain protein of the macrophage colony-stimulating factor 1 receptor (MCSF-1R) inhibited the interaction between pure IL-34 and the monocyte *in vitro*. Additionally, the increase in monocyte vitality brought by IL-34 does not rely on colony-stimulating factor 1 (CSF-1), a second ligand of the CSF-1R (1, 2, 4). Although IL-34 and MCSF-1 exhibit comparable biological activity, myeloid cells respond differently to stimulation by IL-34 or MCSF-1, as evidenced by proinflammatory chemokines production and cytokines (5, 6). The interaction of MCSF-1R with IL-34 has been hypothesized to trigger the activation of autophagy and caspase signaling pathways in monocytes. This activation results in macrophage differentiation and polarization driven by IL-34 rather than CSF-1 (5). Differences in the signaling of CSF-1 and IL-34 have been attributed to differences in the affinity of MCSF-1R binding, hydrophobic/hydrophilic binding character, and differences in the binding pockets (5, 7). However, the molecular processes underlying these disparities must be investigated further in future research.

In 2010, Baudhuin et al. and colleagues published the first research on IL-34’s pathogenic role in malignant giant cell tumors (GCT) (8). GCT is a benign (noncancerous) bone tumor that is rich in osteoclasts, a cell type with a macrophage origin. Previous research suggested that CSF-1 stimulation *via* CSF-1R was necessary for osteoclast formation. Afterward, IL-34 was found to be abundantly expressed inside GCT and can promote mouse and human osteoclastogenesis *via* activating CSF-1R independently of CSF-1 (8, 9). Analysis of tumor-infiltrating immune cells also found a relation between IL-34 expression and type-2 immunosuppressive macrophages in different cancer types (4, 10–12). In patients with large B-cell lymphoma, IL-34 recruits monocytes, resulting in an increased macrophage ratio in the tissues and a poor prognosis (13) In the context of treatment failure, evidence that the TME plays a critical role in progression, metastasis, and therapeutic resistance *via* its interactions with cancer cells is increasing (11, 14). The tumor location of cancer cells that are resistant to treatment has a considerable number of immunosuppressive M2, which inhibits the immunological response against cancer cells (9, 15).

Blockade of immune checkpoints is an intriguing strategy that might potentially stimulate therapeutic anticancer activity. However, malignancies often develop immunological resistance to tumor antigen-specific T cells; hence, treatment outcomes in the clinic are frequently limited. Substantial evidence has shown that IL-34, a cytokine first discovered to control the function and survival of monocytes/macrophages (1, 4), is overexpressed in a wide variety of cancers, where it controls various tumor cell functions (16–18). Recent findings about understanding the activities against ICIs have shown that IL-34 helps malignancies evade the immune system, boosts immunosuppression, and reduces the efficacy of ICIs. In this review, we provide an overview of the current knowledge of the function and role of IL-34 in mediating tumor immunological resistance to ICIs in cancer.

## 2 Immune checkpoint inhibitors

The groundbreaking discovery of ICIs was a significant step forward in the field of immuno-oncology. Cancer cells can circumvent immunosurveillance and advance by various methods, one of which is the activation of immune checkpoint pathways, which inhibit antitumor immune responses. ICIs increase the immune-mediated clearance of tumor cells, revitalize anticancer immune responses by breaking inhibiting signaling pathways, and result in the eradication of cancer cells (19, 20).

**Figure 1:**
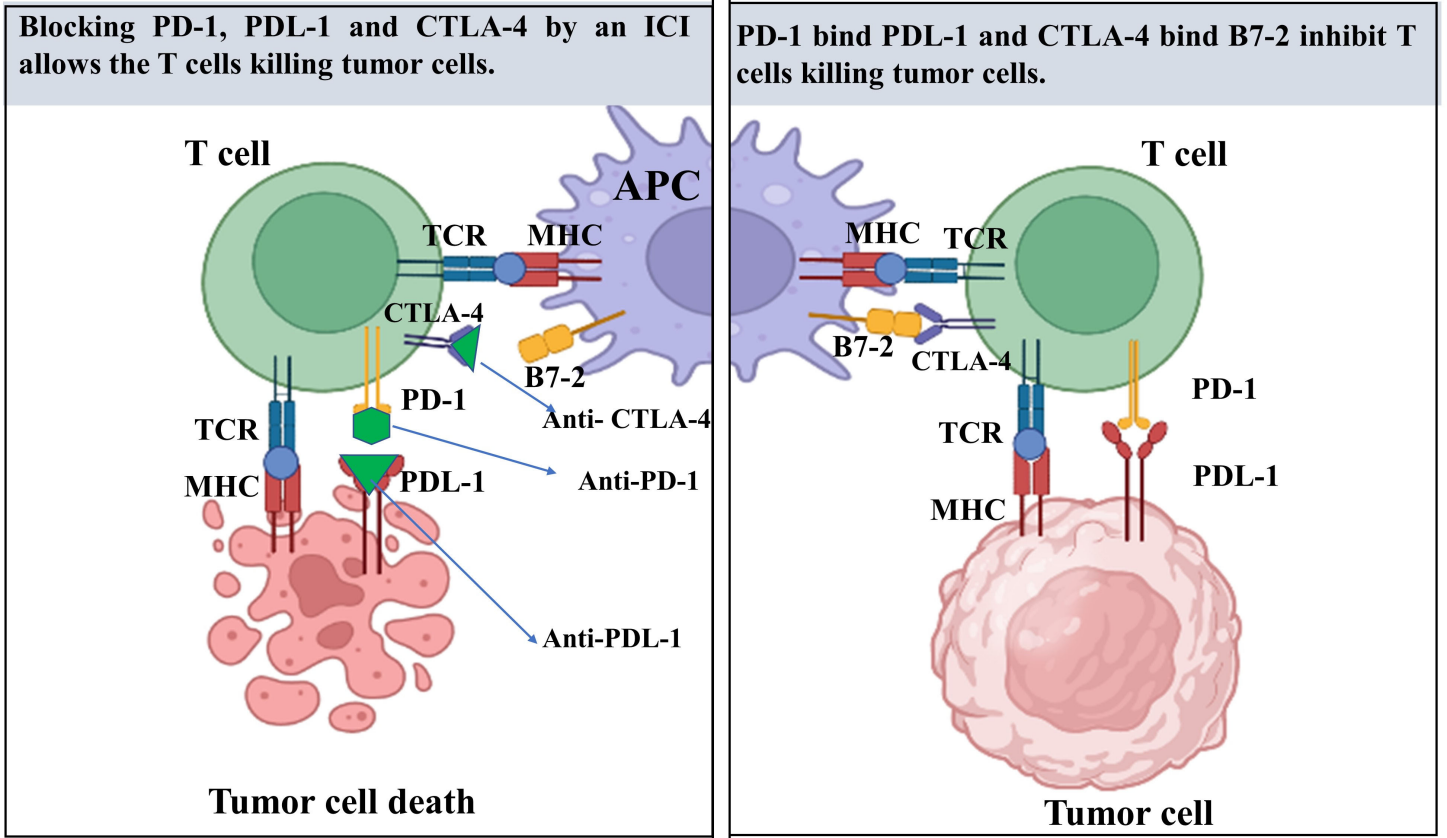
A review of the mechanism of immune checkpoint inhibitors (ICIs) in tumor therapy. T cells recognize antigen-presenting cells (APC), which are primarily dendritic cells (DCs), as well as tumor cells through T cell receptors (TCR). This molecule is responsible for recognizing fragments of antigen known as peptides that are presented to the cell surface of major histocompatibility complex (MHC) molecules. MHC present particular antigens at the cell surface for T-cell receptor (TCR) to becomes active. Co-stimulatory signals are required for T cell activation through the interaction between the CD28 receptor on T cells and the B7 ligands on APC. On T cells, the cytotoxic T-lymphocyte-associated protein 4 (CTLA-4), a co-inhibitory receptor, has been expressed and translocates to the cell membrane after T cell activation, where it competes with CD28 for ligand binding. Consequently, CTLA-4 functions largely during the first phases of cancer growth. in later phase, tumor cells in the tumor microenvironments (TME) release programmed death-ligand 1 (PD-L1), which binds to programmed cell death protein 1(PD-1) receptors on T cells, resulting in a co-inhibitory signal that inhibits T cell function.

Immune checkpoint molecules are inhibitory pathways that the immune system adopts to prevent undesirable self-immune responses (21). T lymphocytes, the main players in cell-mediated immunity, target tumor cells that express tumor-specific antigens in the TME (22). These antigens are displayed on the cell surface by major histocompatibility complex molecules, allowing T lymphocytes to recognize them (23). On the surface of activated T cells, immune inhibitory receptors are expressed including PD-1 and CTLA-4 (21). The checkpoint molecules block T-cell activation by sending a “STOP” signal to their host T cells after binding to their specific ligands. By expressing ligands, such as PD-L1, which are identified by the PD-1 T cell receptor, numerous tumor cells can evade T cell-mediated death. Blocking antibodies that interfere with these receptor–ligand interactions between tumor cells (or antigen-presenting cells) and T cells might alleviate the patch on T cells, hence releasing T cell-mediated anticancer effects. Undoubtedly, antibodies that inhibit checkpoints have been shown to be particularly effective against a wide range of cancers. In 2011, the USFDA has approved the first ICI therapy (Ipilimumab) specifically for metastatic melanoma treatment (24), leading to potential response rate regardless of surgery or targeted therapy (25). In the past few years, various studies for immune blockage mainly in renal cell carcinoma, lung cancer, pancreatic cancer (PC) and prostate cancer have yielded promising results, indicating the considerable potential for such a therapy strategy (14, 26, 27) (**Figure 1**). Although PD-L1 is predominantly expressed on tumor cells, it may also be expressed on antigen-presenting cells; hence, the PD-1/PD-L1 T cell inhibitory mechanism operates throughout several stages of an immune response. PD-L1 and PD-L2 are members of the B7-H1 and B7-DC subfamilies, respectively. Therefore, enhancing the anticancer efficacy of ICIs in patients with cancer and reversing the immunosuppressive phenotype are crucial.

During the genesis of cancer, the TME gains immunosuppression and resistance to cancer therapy. Recently, ICIs for cancer has gained popularity as a therapy following the three primary traditional tumor therapies (surgery, radiation, and chemotherapy) to combat the TME. Blocking antibodies, particularly PD-1 and CTLA-4, have shifted the paradigm of cancer therapy; however, many patients with cancer do not respond and develop resistance to ICIs. In addition to its vital function in tumors, IL-34 has been identified as a factor that biases macrophages toward immunosuppression, leading to increased tumor evasion and progression (28). ICIs alone are not sufficient for effective treatment. However, the combination of ICIs with other newly discovered targetable biomarkers can have the potential to reverse the immunosuppressive state in the TME and may represent a promising approach for future treatment strategies.

## 3 IL-34 and ICIs in various cancer types

The immunotherapies that target ICI molecules, e.g., CTLA 4 (29, 30), and PD-1 (31, 32), have contributed to the achievement of persistent responses in the treatment of cancer. Nevertheless, 25% of patients with melanoma that showed objective responses to ICIs acquire resistance to the therapy and suffer from the development of their disease and death (33). Numerous studies have proposed several mechanisms for immune system resistance at the cellular and molecular levels. These mechanisms include weakened T cell infiltration and activation at the TME, epigenetic alterations in cancer cells that impair IFN‐γ signaling, and local TME immunosuppression (21, 34, 35). In this regard, important roles in acquired and innate resistance to ICIs have been attributed to the infiltration of the TME with immunosuppression cells, such as M2-biased TAMs, regulatory T cells (Tregs), and myeloid-derived suppressor cells (MDSCs), as well as numerous inflammatory and metabolic mediators such as arginase1 (ARG1), prostaglandin E2 (PGE2), and indoleamine 2,3-dioxygenase (IDO) (36–38). In many instances, the soluble molecules secreted by the tumor cells are responsible for the formation and maintenance of the immunosuppression TME (39). In light of this, focusing on these parameters would make it possible to alleviate immunosuppression and increase immunotherapeutic responses (40, 41).

Recently, IL-34 and its receptor, CSF-1R, has drawn strong interest as crucial proteins controlling the survival, function, and proliferation of M2-biased TAMs, which are distinguished by their immunosuppressive activities (42, 43). In several cancers, notably melanoma, PC, and hepatocellular carcinoma, the CSF-1/CSF-1R axis has been associated with the emergence of resistance to PD-1/PD-L1 suppression (44–48). In addition to being activated by CSF-1, the CSF-1R receptor can also be activated differently by binding IL-34 as the second ligand (1). Although CSF-1 and IL-34 have the same receptor and have a comparable effect *in vitro* on myeloid cells, the two cytokines bind to distinct pockets within the extracellular immunoglobulin (Ig)-like domains of CSF-1R, resulting in various activation pathways of CSF-1R (49). An important distinction between CSF-1 and IL-34 is that IL-34 is selectively expressed in the skin and brain, whereas CSF-1 is physiologically widely dispersed throughout the body (50). Even though it is selectively expressed, IL-34 may be released by tumor cells, and it plays a crucial role in the development of tumors (1, 12, 50–54) and their resistance to chemotherapy and molecularly targeted treatment (15, 55).

**Figure 2:**
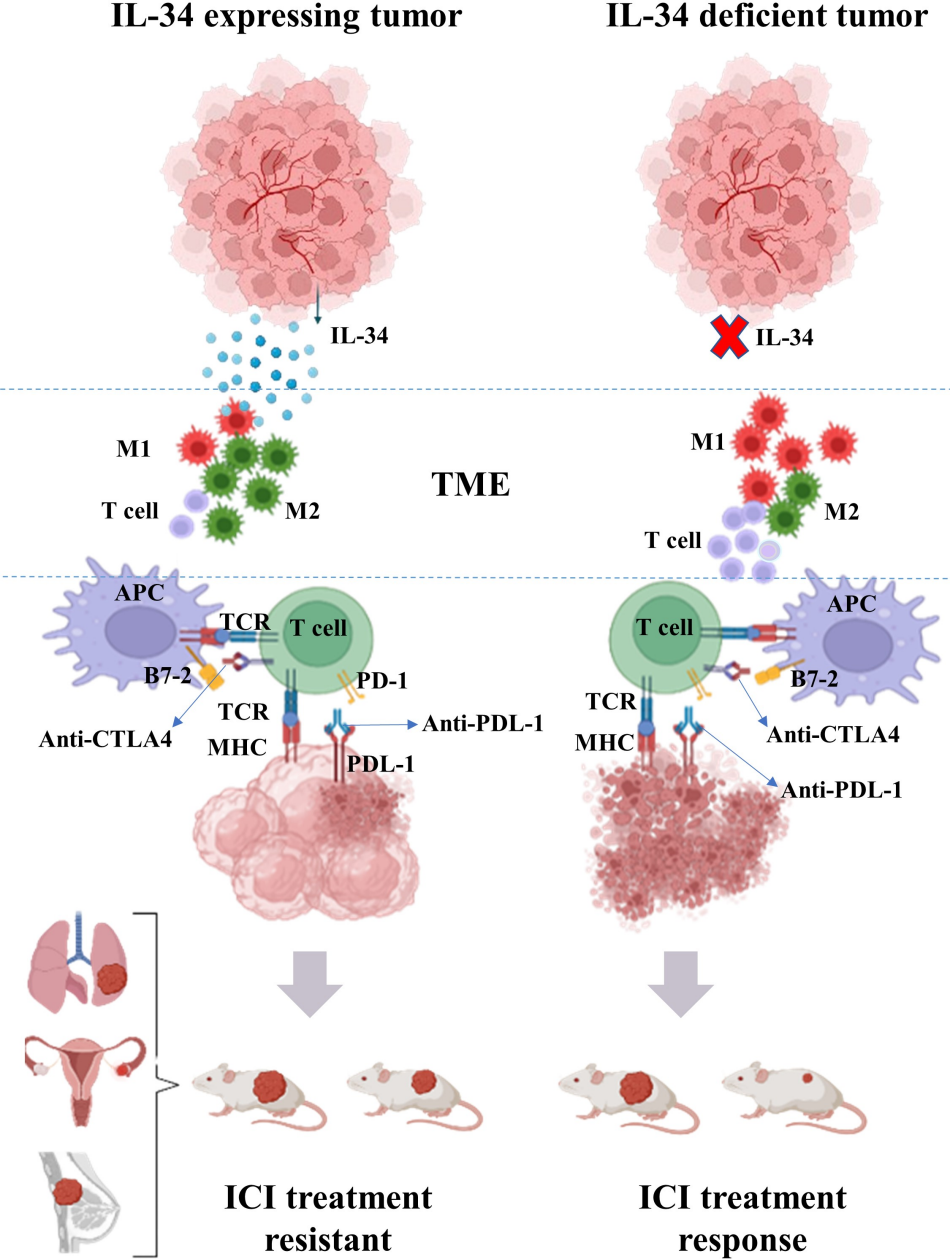
Overview of the effect of IL-34 on the efficacy of immune checkpoint inhibitors (ICIs) in the treatment of cancer. Tumor-derived IL-34 can modulate the tumor microenvironments (TME) leading to lower frequency of T cell and increases in the ratio of M2-biased macrophages to M1-biased macrophages in TME. Conjunction with an ICI to treat malignancies, a neutralizing antibody directed against IL-34 increase the therapeutic benefits of the inhibitor.

Consistent with its immunosuppressive properties, IL-34 expression in tumors is related to lower cellular frequencies (CD4^+^, CD8^+^ T cells, and M1-biased macrophage) and molecular (various chemokines and cytokines) effector frequencies in the TME (11, 56). Using a neutralizing antibody against IL-34 improved ICI’s therapeutic advantages in combinational therapy models involving a patient-derived xenograft model (57). Therefore, cancer therapy may gain a potentially game-changing new option if IL-34 inhibitors can be used to limit protumorigenic effects and ICI resistance (**Table 1** and **Figure 2**).

**Table 1:** The effect of IL-34 on cancer-related ICIs.

Type of cancer	ICIs treatment	IL-34 effect	Ref
Ovarian cancer	Anti-PD-1	Cancer-derived IL-34 inhibits antitumor T cell-mediated immunity, impacting the effectiveness of PD-1 blocking treatment.	(60)
Colon cancer	Anti-PD-1 Anti-CTLA-4	IL-34KO inhibits tumor progression, increases the M1 subset while decreasing the M2 subset, and increases the production of inflammatory and pro-inflammatory cytokines.	(60)
Breast cancer			
Malignant melanoma	Anti-PD-1	Nivolumab-resistant metastatic melanoma showed an increase in the expression of IL-34 associated with higher M2 macrophage infiltration.	(78)
Lung cancer	Anti-PD-1	IL-34 blockade with anti-PD-1 antibodies considerably inhibited tumor development with significant immune cell infiltration.	(60)

### 3.1 Ovarian cancer

Ovarian cancer is the third most frequent gynecological malignancy globally, yet it has the greatest fatality rate among these tumors. Immunotherapy response rates for patients with ovarian cancer remain to be modest, although ICI therapy is developing quickly (58, 59). In ovarian cancer, to assess the effects of IL-34 on ICI therapy, Hama et al. (60) conducted their study with the mouse ovarian tumor cell line-HM-1, which produces a considerable amount of IL-34. Only IL-34^KO^ HM-1 tumor reacted to PD-1 blockage exhibiting reduced tumor sizes than the control IgG therapy. Furthermore, PD-1 blockade had a minor effect in a mock HM-1, suggesting that the blockade of PD-1 is most effective in ovarian cancer without IL-34 (60). Moreover, the IL-34^KO^ HM-1 tumor exhibited a large infiltrating of CD4^+^ T and CD8^+^ cells; these data indicate the increase in T cell infiltration in the IL-34^KO^ HM-1 tumor is not dependent on PD-1 inhibition. In addition, quantitative PCR analysis demonstrated that IL-34^KO^ HM-1 tumors had elevated levels of IFN-γ and tumor necrosis factor-alpha (TNF-α) (60). These results suggest that IL-34 excreted by cancer cells may inhibit antitumor T cell-mediated immunity, which appears to have influenced the effectiveness of PD-1 blocking treatment.

The production of T cell chemoattractants Cxcl11, Cxcl10, and Cxcl9 is stimulated by IFN-γ (61). Cxcl9 expression considerably increased in tumors of IL-34^KO^ HM-1, which had been treated with an anti-PD-1 antibody (60). By interacting with its receptor Cxcr3, Cxcl9 has been demonstrated to enhance the activity of T cell killers considerably (62), which may explain why PD-1 inhibition was more effective against IL-34^KO^ HM-1 tumors.

To assess *in vivo* the therapeutic advantages of IL-34 blockage in combination with ICIs, IL-34-expressed mock HM-1 cells were implanted into B6C3F1 mice; subsequently treated with anti-PD-1, a combination of anti-PD-1 and IL-34 antibodies, or IgG as a control. Compared with the control group, PD-1 inhibition alone had a negligible effect on tumor growth 14 days after therapy. By contrast, PD-1 inhibition combined with an anti-IL-34 antibody remarkably suppressed tumor growth and weight (60). Comparing the TME cellular components in each group revealed that inhibiting IL-34 increased CD8^+^ T cell infiltration but had no effect on CD4^+^ T cells. Additionally, combined therapy demonstrated a small reduction in CD11b^+^F4/80^+^ cells (60). All these results interpret the broad therapeutic effect of IL-34 blockage inside the TME. In the same context, several recent studies have shown that the blockage of CSF-1R increases the antitumor response to anti-PD-1 therapy in a wide range of tumor types including ovarian cancer (63–66). Comparing the anticancer effectiveness of IL-34 blockage to that of CSF-1R blockage *in vivo* under the same conditions, reveal a similar effect when paired with anti-PD-1 therapy (48, 67–71). Overall, these findings suggest that blocking IL-34 or its receptor, CSF-1R, can potentially increase the effectiveness of anti-PD-1 treatment.

### 3.2 Colon and breast cancer

Colon cancer is the most common cancer worldwide and one of the top three malignancies in the USA in terms of occurrence and fatality (72). However, breast cancer remains the most common cancer-related cause of burden disease for women, affecting one out of 20 worldwide and up to one in every eight in developed countries (4, 73). Many studies have reported that IL-134 overexpression is conversely correlated with poor survival and prognosis in patients with colon and breast cancer (43). Concerning cancer immunotherapy, CT26 colon cancer cells are sensitive to ICIs, such as anti-CTLA-4 and anti-PD-1 antibodies (74, 75). However, the efficacy of ICIs in this cell line is dramatically diminished by IL-34 overexpression (60, 76, 77). To determine how tumor cell-derived IL-34 blocks the ICIs in colon and breast cancer, researchers used CRISPR-Cas9 technology to create IL-34-deficient CT26 and 4T1 cell lines, respectively. The IL-34^KO^ CT26 cell line was induced to express IL-34 and generate an IL-34-overexpressed cell line (IL-34^OE^ CT26). The tumor cells were subsequently transplanted into Syngenic BALB/c mice, which were then treated with either anti-PD-1 or IgG as control. The IL-34^KO^ tumors responded better to anti-PD-1 antibody therapy, as evidenced by reduced tumor sizes in comparison to the control IgG; however the favorable effects of PD-1 blocking were canceled out by IL-34, which is produced by tumor cells (60).

Next-generation sequencing and gene ontology analysis determined which set of genes was responsible for the greatest antitumor effectiveness within the study group. The group that has been infected with IL-34^KO^ CT26 and treated with anti-PD-1 exhibited a greater number of immune cell response clusters. These immune cell response clusters included the signaling pathway of T cell receptors, antigen presentation and processing, and cytokine–cytokine receptor interactions. Moreover, many genes related to an accumulation of T cells (Cd8a, Cd3e, Cd4), inflammations (Cxcl9, Tnf, Ifng), and a subset of M1-macrophage (Ciita, Nos2, Cd86) have elevated in anti-PD-1 antibody-treated IL-34^KO^ CT26 tumor. By contrast, gene expressions of the M2-macrophage subset (Arg1, Mrc1, and Chi3l3) were lower in IL-34^KO^ CT26 tumors treated with anti-PD-1 than in IL-34^OE^ CT26 tumors (60). In this context, molecular evidence suggests that IL-34^KO^ inhibits the progression of tumors by inducing an elevation in the M1 subset and a reduction in the M2 subset markers.

In the therapeutic models’ cellular components of the TME, IL-34 was shown to alter the cellular profiles and the inflammatory state of the TME in the tumor (10, 56, 78). An IL-34^KO^ CT26 tumor cell model was conducted to determine if tumor-derived IL-34 contributes to TME M1- and M2-predominant macrophage populations. The results showed that the number of tumor-infiltrating Nos2^+^ M1-biased macrophages increased whereas the number of Arg1^+^ M2-biased macrophages decreased (10, 51, 60). CSF-1R inhibition causes a substantial decrease in tumor-associated macrophages (79, 80). In the same context when treated with a PD-1 inhibitor, the 4T1 and CT26 tumor cell models demonstrated a considerably increased production of inflammatory and pro-inflammatory cytokines. Additionally, the number of M1-biased macrophages that infiltrated the tumor were shown to be higher in the IL-34^KO^ 4T1 tumor (60). These results imply that IL-34 produced by tumors may inhibit the growth of M1-biased macrophages and inhibit the formation of the anti-inflammatory milieu. To understand further the correlation between IL-34 and ICIs resistance, the combination of anti-PD-1 and anti-CTLA-4 with or without anti-IL-34 antibodies was added to IL-34^OE^ CT26 tumors. The combined therapy of CTLA-4 and anti-PD-1 without anti-IL-34 antibodies resulted in considerable suppression of tumors, whereas anti-CTLA-4 and anti-PD-1 combined with anti-IL-34 antibodies resulted in a more dramatic suppression of tumors (60). Overall, these findings suggest that IL-34 inhibits the efficiency of ICIs not only by competing with accumulations of T-cells but also by changing the inflammatory state of TME through an imbalance of polarization in the M1 and M2 macrophage populations. Additionally, inhibiting IL-34 with particular antibodies inverts ICI resistance by restoring the inflammatory circuitry inside the TME.

### 3.3 Lung cancer

Lung cancer is a common leading cause of death among patients with cancer worldwide. The mortality rate of lung cancer remains high despite breakthroughs in treatment during the previous decade, including targeted medications, diagnostics, staging, surgical procedures, and chemotherapy and radiation (81). The expression of IL-34 and lung cancer progression is still under debate; some previous studies have reported that IL-34 overexpression is related to the poor survival and prognosis of patients with lung cancer. By contrast, Zhendong et al. found that the loss expression of IL-34 correlated with poor prognosis of patients with lung adenocarcinoma (LUAD) (56, 82, 83). Recently, ICIs have shown promise in treating majority of lung cancer subtypes, including nonsmall cell lung cancer (adenocarcinoma, squamous cell carcinoma, and large cell carcinoma) (84, 85). LUAD is a major type of lung cancer with a five-year overall survival rate of fewer than 15% because majority of patients are detected at a late stage during their initial visit (86). In immunotherapy, IL-34 blockade has potential therapeutic effects in LUAD. In clinical settings, the patient-derived xenograft (PDX) model of human primary lung cancer tissues was injected into HuNSG mice. The humanized PDX mice model revealed that PD-L1 and IL-34 were highly expressed in LUAD, which was then treated with anti-PD-1 antibodies alone or anti-PD-1 antibodies and IL-34 antibodies in combination. Consequently, PD-1 blockage alone had an insufficient response, whereas the combination of both antibodies considerably inhibited tumor development (60). This result shows that the combined treatment was successful in releasing a powerful anticancer effect. Moreover, a substantial amount of immune cell infiltration was observed in the tumor that responded to combination treatment (60). Therefore, IL-34 contributes to the development of ICI resistance in human cancer, and inhibiting IL-34 can restore the therapeutic effect of ICIs.

### 3.4 Melanoma

Melanoma is the most lethal type of skin cancer, and its prevalence has increased dramatically in recent decades (87, 88). Several studies have recently revealed specific biomarkers that may lead to aggressive behavior in malignant melanoma cells. Giricz, et al. found that high IL-34 expression was associated with resistance and poor prognosis in patients with malignant melanoma (55). In the treatment of melanoma, the use of ICIs that target immune checkpoint molecules has contributed to the achievement of sustained responses (89). Nevertheless, after a median follow-up of 21 months, 25% of patients with melanoma who demonstrated objective responses to PD-1 blockade acquired resistance and suffered disease progression (90). Immune resistance mechanisms in melanoma have mostly remained unexplored. Previous studies in melanoma tissues found a link between genetic changes and acquired ICI resistance (91–94) such as the lack of beta-2-microglobulin (95) or anomalies in the IFN pathways (96). Under normal conditions, IL-34 expression is restricted to the brain and the skin, where it regulates the development, biology, and function of Microglia and Langerhans cells, respectively (97, 98).

The most recent data in a case-control study found that a patient with refractory melanoma exhibited elevated IL-34 expression (77). These findings are consistent with previous studies that reported a correlation between IL-34 expression and tumor metastasis, progression, and resistance to therapy (8, 12, 51, 99, 100). Tissue from a patient with Nivolumab-resistant metastatic melanoma showed a remarkable increase in expression of IL-34 compared with primary site melanoma tissues, and this upsurge in expression was demonstrated to be connected with greater frequencies of CD163, an M2 macrophage marker (77). This result is consistent with previous studies that provided evidence that IL-34 plays a key role in the induction of M2-polarized macrophages, which have immunosuppressive effects (101). Tumor-derived IL-34 enhances tumor-associated M2-polarized macrophages that exhibit immunosuppression and promote tumor growth (15). Compared with primary melanoma, elevated IL-34 expression is associated with increased CD163^+^ (an M2-polarization marker) macrophages in refractory metastatic melanoma (15). According to the Human Protein Atlas (http://www.proteinatlas.org ) (102), higher IL-34 expression is related with poor prognosis in melanoma, as shown by the Kaplan–Meier analysis of overall survival (77). As a result, many studies are now using experimental animal models to determine whether or not inhibiting IL-34 in IL-34-producing melanomas may help overcome the therapeutic resistance issues.

## 4 Future perspectives

Widespread cancer types may benefit from the immunoregulatory activities of IL-34. An increase in tumorigenesis is associated with the overexpression of IL-34, which has been demonstrated to play a role in the resistance, progression, and overall survival of patients. Despite the many advantages of ICIs, several challenges about ICI resistance have not been addressed. IL-34 may be responsible for inhibiting the therapeutic benefits of ICIs, according to new findings. To assess ICIs effectively, having a comprehensive understanding of the potential benefits and risks of IL-34 dysregulation in the TME is vital. Therefore, clinical trials based on robust preclinical evidence, as well as extensive translational research to explain the optimal dose and administration, potential side effects with safety, and the mechanisms of IL-34’s effects in combination with ICIs, are essential to further treatment approaches for various types of cancer.

## 5 Conclusion

Since its discovery, IL-34 has been the subject of studies regarding how it affects immune responses. Furthermore, IL-34 and its functional receptors activate intracellular pathways that are involved in the development and progression of many different types of cancer. Here, studies suggesting that IL-34 may play a role in the development of therapeutic resistance to tumor-ICI therapies were summarized. IL-34 limits the effectiveness of ICIs not only by interfering with T-cell attraction but also by altering the inflammatory situation of the TME through polarization imbalances of the M1 and M2 macrophage populations. The combination of IL-34 inhibition with ICIs has raised its profile as a potentially essential treatment option for patients with cancer in the future.

## Author contributions

The review was designed and revised by FA, MS, YAA, Al-Azab M, AA, MA and AR. FA and MS conducted the literature collection. FA and YAA draw the figures and CZ provided guidance and revised this manuscript. Correspondence MS and CZ. Funding and correspondence; CZ. All authors contributed to the article and approved the submitted version.

## References

[1] Lin H Lee E Hestir K Leo C Huang M Bosch E. Discovery of a cytokine and its receptor by functional screening of the extracellular proteome. Science. (2008) 320(5877):807–11. doi: 10.1126/science.1154370 18467591

[2] Ma X Lin WY Chen Y Stawicki S Mukhyala K Wu Y. Structural basis for the dual recognition of helical cytokines IL-34 and CSF-1 by CSF-1R. Structure. (2012) 20(4):676–87. doi: 10.1016/j.str.2012.02.010 22483114

[3] Sjöstedt E Zhong W Fagerberg L Karlsson M Mitsios N Adori C. An atlas of the protein-coding genes in the human, pig, and mouse brain. Science (2020) 367(6482). doi: 10.1126/science.aay5947 32139519

[4] Monteleone G Maresca C Colella M Pacifico T Congiu D Troncone E. Targeting IL-34/MCSF-1R axis in colon cancer. Front Immunol (2022) 13:917955. doi: 10.3389/fimmu.2022.917955 35837402 PMC9273844

[5] Boulakirba S Pfeifer A Mhaidly R Obba S Goulard M Schmitt T. IL-34 and CSF-1 display an equivalent macrophage differentiation ability but a different polarization potential. Sci Rep (2018) 8(1):1–11. doi: 10.1038/s41598-017-18433-4 29321503 PMC5762882

[6] Nakamichi Y Udagawa N Takahashi N. IL-34 and CSF-1: similarities and differences. J Bone mineral Metab (2013) 31(5):486–95. doi: 10.1007/s00774-013-0476-3 23740288

[7] Liu H Leo C Chen X Wong BR Williams LT Lin H. The mechanism of shared but distinct CSF-1R signaling by the non-homologous cytokines IL-34 and CSF-1. Biochim Biophys Acta (BBA)-Proteins Proteomics. (2012) 1824(7):938–45. doi: 10.1016/j.bbapap.2012.04.012 PMC3372767 22579672

[8] Baud'Huin M Renault R Charrier C Riet A Moreau A Brion R. Interleukin-34 is expressed by giant cell tumours of bone and plays a key role in RANKL-induced osteoclastogenesis. J pathology. (2010) 221(1):77–86. doi: 10.1002/path.2684 20191615

[9] Otsuka R Wada H Seino K-i eds. IL-34, the rationale for its expression in physiological and pathological conditions. In: Seminars in immunology. 54, 101517. Academic Press: Elsevier. 10.1016/j.smim.2021.101517 34774392

[10] Zins K Heller G Mayerhofer M Schreiber M Abraham D. Differential prognostic impact of interleukin-34 mRNA expression and infiltrating immune cell composition in intrinsic breast cancer subtypes. Oncotarget (2018) 9(33):23126. doi: 10.18632/oncotarget.25226 29796177 PMC5955405

[11] Wang Z Wang F Ding X-Y Li T-E Wang H-Y Gao Y-H. Hippo/YAP signaling choreographs the tumor immune microenvironment to promote triple negative breast cancer progression *via* TAZ/IL-34 axis. Cancer Letters. (2022) 527:174–90. doi: 10.1016/j.canlet.2021.12.016 34929335

[12] Zhou SL Hu ZQ Zhou ZJ Dai Z Wang Z Cao Y. miR-28-5p-IL-34-macrophage feedback loop modulates hepatocellular carcinoma metastasis. Hepatology. (2016) 63(5):1560–75. doi: 10.1002/hep.28445 26754294

[13] Noyori O Komohara Y Nasser H Hiyoshi M Ma C Pan C. Expression of IL-34 correlates with macrophage infiltration and prognosis of diffuse large b-cell lymphoma. Clin Transl Immunol (2019) 8(8):e1074. doi: 10.1002/cti2.1074 PMC6691654 31417675

[14] Wang S Li Y Xing C Ding C Zhang H Chen L. Tumor microenvironment in chemoresistance, metastasis and immunotherapy of pancreatic cancer. Am J Cancer Res (2020) 10(7):1937–53. PMC7407356 32774994

[15] Baghdadi M Wada H Nakanishi S Abe H Han N Putra WE. Chemotherapy-induced IL34 enhances immunosuppression by tumor-associated macrophages and mediates survival of chemoresistant lung cancer CellsAn importance of IL34 in cancer chemoresistance. Cancer Res (2016) 76(20):6030–42. doi: 10.1158/0008-5472.CAN-16-1170 27550451

[16] Lin W Xu D Austin CD Caplazi P Senger K Sun Y. Function of CSF1 and IL34 in macrophage homeostasis, inflammation, and cancer. Front Immunol (2019) 10:2019. doi: 10.3389/fimmu.2019.02019 31552020 PMC6736990

[17] Freuchet A Salama A Remy S Guillonneau C Anegon I. IL-34 and CSF-1, deciphering similarities and differences at steady state and in diseases. J Leukocyte Biol (2021) 110(4):771–96. doi: 10.1002/JLB.3RU1120-773R 33600012

[18] Franzè E Marafini I Troncone E Salvatori S Monteleone G. Interleukin-34 promotes tumorigenic signals for colon cancer cells. Cell Death discovery. (2021) 7(1):1–7. doi: 10.1038/s41420-021-00636-4 PMC8448832 34535634

[19] Haslam A Prasad V. Estimation of the percentage of US patients with cancer who are eligible for and respond to checkpoint inhibitor immunotherapy drugs. JAMA network Open (2019) 2(5):e192535–e. doi: 10.1001/jamanetworkopen.2019.2535 PMC6503493 31050774

[20] Darvin P Toor SM Sasidharan Nair V Elkord E. Immune checkpoint inhibitors: recent progress and potential biomarkers. Exp Mol Med (2018) 50(12):1–11. doi: 10.1038/s12276-018-0191-1 PMC6292890 30546008

[21] Pardoll DM. The blockade of immune checkpoints in cancer immunotherapy. Nat Rev Cancer. (2012) 12(4):252–64. doi: 10.1038/nrc3239 PMC4856023 22437870

[22] Rivoltini L Carrabba M Huber V Castelli C Novellino L Dalerba P. Immunity to cancer: Attack and escape in T lymphocyte–tumor cell interaction. Immunol Rev (2002) 188(1):97–113. doi: 10.1034/j.1600-065X.2002.18809.x 12445284

[23] Renkvist N Castelli C Robbins PF Parmiani G. A listing of human tumor antigens recognized by T cells. Cancer Immunol Immunother (2001) 50(1):3–15. doi: 10.1007/s002620000169 11315507 PMC11036832

[24] Lipson EJ Drake CG. Ipilimumab: An anti-CTLA-4 antibody for metastatic MelanomaIpilimumab for metastatic melanoma. Clin Cancer Res (2011) 17(22):6958–62. doi: 10.1158/1078-0432.CCR-11-1595 PMC3575079 21900389

[25] Paddock LE Lu SE Bandera EV Rhoads GG Fine J Paine S. Skin self-examination and long-term melanoma survival. Melanoma Res (2016) 26(4):401–8. doi: 10.1097/CMR.0000000000000255 26990272

[26] Sharon E Streicher H Goncalves P Chen HX. Immune checkpoint inhibitors in clinical trials. Chin J cancer. (2014) 33(9):434. doi: 10.5732/cjc.014.10122 25189716 PMC4190433

[27] Morrison AH Byrne KT Vonderheide RH. Immunotherapy and prevention of pancreatic cancer. Trends Cancer. (2018) 4(6):418–28. doi: 10.1016/j.trecan.2018.04.001 PMC6028935 29860986

[28] Al-Shaebi F Wenzhang L Hezam K Almezgagi M Wei L. Recent insights of the role and signalling pathways of interleukin-34 in liver diseases. Int Immunopharmacology. (2020) 89:107023. doi: 10.1016/j.intimp.2020.107023 33129098

[29] Schadendorf D Hodi FS Robert C Weber JS Margolin K Hamid O. Pooled analysis of long-term survival data from phase II and phase III trials of ipilimumab in unresectable or metastatic melanoma. J Clin Oncol (2015) 33(17):1889. doi: 10.1200/JCO.2014.56.2736 25667295 PMC5089162

[30] Eroglu Z Kim DW Wang X Camacho LH Chmielowski B Seja E. Long term survival with cytotoxic T lymphocyte-associated antigen 4 blockade using tremelimumab. Eur J Cancer. (2015) 51(17):2689–97. doi: 10.1016/j.ejca.2015.08.012 PMC4821004 26364516

[31] Robert C Long GV Brady B Dutriaux C Maio M Mortier L. Nivolumab in previously untreated melanoma without BRAF mutation. New Engl J Med (2015) 372(4):320–30. doi: 10.1056/NEJMoa1412082 25399552

[32] Hamid O Robert C Daud A Hodi FS Hwu W-J Kefford R. Safety and tumor responses with lambrolizumab (anti–PD-1) in melanoma. New Engl J Med (2013) 369(2):134–44. doi: 10.1056/NEJMoa1305133 PMC4126516 23724846

[33] Schoenfeld AJ Hellmann MD. Acquired resistance to immune checkpoint inhibitors. Cancer Cell (2020) 37(4):443–55. doi: 10.1016/j.ccell.2020.03.017 PMC7182070 32289269

[34] O'Donnell JS Long GV Scolyer RA Teng MW Smyth MJ. Resistance to PD1/PDL1 checkpoint inhibition. Cancer Treat Rev (2017) 52:71–81. doi: 10.1016/j.ctrv.2016.11.007 27951441

[35] Ribas A. Adaptive immune resistance: How cancer protects from immune AttackAdaptive immune resistance. Cancer discovery. (2015) 5(9):915–9. doi: 10.1158/2159-8290.CD-15-0563 PMC4560619 26272491

[36] Ugel S De Sanctis F Mandruzzato S Bronte V. Tumor-induced myeloid deviation: When myeloid-derived suppressor cells meet tumor-associated macrophages. J Clin Invest (2015) 125(9):3365–76. doi: 10.1172/JCI80006 PMC4588310 26325033

[37] Prima V Kaliberova LN Kaliberov S Curiel DT Kusmartsev S. COX2/mPGES1/PGE2 pathway regulates PD-L1 expression in tumor-associated macrophages and myeloid-derived suppressor cells. Proc Natl Acad Sci (2017) 114(5):1117–22. doi: 10.1073/pnas.1612920114 PMC5293015 28096371

[38] Kumar V Patel S Tcyganov E Gabrilovich DI. The nature of myeloid-derived suppressor cells in the tumor microenvironment. Trends Immunol (2016) 37(3):208–20. doi: 10.1016/j.it.2016.01.004 PMC4775398 26858199

[39] Binnewies M Roberts EW Kersten K Chan V Fearon DF Merad M. Understanding the tumor immune microenvironment (TIME) for effective therapy. Nat Med (2018) 24(5):541–50. doi: 10.1038/s41591-018-0014-x PMC5998822 29686425

[40] Ghirelli C Hagemann T. Targeting immunosuppression for cancer therapy. J Clin Invest (2013) 123(6):2355–7. doi: 10.1172/JCI69999 PMC3668808 23728169

[41] Pitt J Marabelle A Eggermont A Soria J-C Kroemer G Zitvogel L. Targeting the tumor microenvironment: Removing obstruction to anticancer immune responses and immunotherapy. Ann Oncol (2016) 27(8):1482–92. doi: 10.1093/annonc/mdw168 27069014

[42] Noy R Pollard JW. Tumor-associated macrophages: from mechanisms to therapy. Immunity. (2014) 41(1):49–61. doi: 10.1016/j.immuni.2014.06.010 25035953 PMC4137410

[43] Poudel M Kim G Bhattarai PY Kim JY Choi HS. Interleukin-34-CSF1R signaling axis promotes epithelial cell transformation and breast tumorigenesis. Int J Mol Sci (2021) 22(5). doi: 10.3390/ijms22052711 PMC7962444 33800170

[44] Zhu Y Yang J Xu D Gao X-M Zhang Z Hsu JL. Disruption of tumour-associated macrophage trafficking by the osteopontin-induced colony-stimulating factor-1 signalling sensitises hepatocellular carcinoma to anti-PD-L1 blockade. Gut. (2019) 68(9):1653–66. doi: 10.1136/gutjnl-2019-318419 30902885

[45] Quaranta V Rainer C Nielsen SR Raymant ML Ahmed MS Engle DD. Macrophage-derived granulin drives resistance to immune checkpoint inhibition in metastatic pancreatic CancerGranulin drives anti–PD-1 therapy resistance in PDAC. Cancer Res (2018) 78(15):4253–69. doi: 10.1158/0008-5472.CAN-17-3876 PMC6076440 29789416

[46] Neubert NJ Schmittnaegel M Bordry N Nassiri S Wald N Martignier C. T Cell–induced CSF1 promotes melanoma resistance to PD1 blockade. Sci Trans Med (2018) 10(436):eaan3311. doi: 10.1126/scitranslmed.aan3311 PMC5957531 29643229

[47] Gyori D Lim EL Grant FM Spensberger D Roychoudhuri R Shuttleworth SJ. Compensation between CSF1R+ macrophages and Foxp3+ treg cells drives resistance to tumor immunotherapy. JCI Insight (2018) 3(11). doi: 10.1172/jci.insight.120631 PMC6124419 29875321

[48] Cannarile MA Weisser M Jacob W Jegg A-M Ries CH Rüttinger D. Colony-stimulating factor 1 receptor (CSF1R) inhibitors in cancer therapy. J immunotherapy cancer. (2017) 5(1):1–13. doi: 10.1186/s40425-017-0257-y PMC5514481 28716061

[49] Kim R Emi M Tanabe K Arihiro K. Tumor-driven evolution of immunosuppressive networks during malignant progression. Cancer Res (2006) 66(11):5527–36. doi: 10.1158/0008-5472.CAN-05-4128 16740684

[50] Wang Y Szretter KJ Vermi W Gilfillan S Rossini C Cella M. IL-34 is a tissue-restricted ligand of CSF1R required for the development of langerhans cells and microglia. Nat Immunol (2012) 13(8):753–60. doi: 10.1038/ni.2360 PMC3941469 22729249

[51] Ségaliny AI Mohamadi A Dizier B Lokajczyk A Brion R Lanel R. Interleukin-34 promotes tumor progression and metastatic process in osteosarcoma through induction of angiogenesis and macrophage recruitment. Int J cancer. (2015) 137(1):73–85. doi: 10.1002/ijc.29376 25471534

[52] Franzè E De Simone V Dinallo V Rizzo A Caprioli F Colantoni A. OC. 01.4: interleukin-34 sustains pro-tumorigenic signals in colon cancer tissue. Digestive Liver Disease. (2017) 2(49):e78. doi: 10.18632/oncotarget.23289 PMC5790474 29423057

[53] Baghdadi M Endo H Takano A Ishikawa K Kameda Y Wada H. High co-expression of IL-34 and m-CSF correlates with tumor progression and poor survival in lung cancers. Sci Rep (2018) 8(1):1–10. doi: 10.1038/s41598-017-18796-8 29323162 PMC5765132

[54] Baghdadi M Ishikawa K Nakanishi S Murata T Umeyama Y Kobayashi T. A role for IL-34 in osteolytic disease of multiple myeloma. Blood advances. (2019) 3(4):541–51. doi: 10.1182/bloodadvances.2018020008 PMC6391661 30782613

[55] Giricz O Mo Y Dahlman KB Cotto-Rios XM Vardabasso C Nguyen H. The RUNX1/IL-34/CSF-1R axis is an autocrinally regulated modulator of resistance to BRAF-V600E inhibition in melanoma. JCI Insight (2018) 3(14). doi: 10.1172/jci.insight.120422 PMC6124424 30046005

[56] Wang Z Zhu J Wang T Zhou H Wang J Huang Z. Loss of IL-34 expression indicates poor prognosis in patients with lung adenocarcinoma. Front Oncol (2021) 11:639724. doi: 10.3389/fonc.2021.639724 34336646 PMC8322957

[57] Han N Jang HY Hama N Kobayashi T Otsuka R Wada H. An optimized protocol for patient-derived xenograft in humanized mice to evaluate the role of IL-34 in immunotherapeutic resistance. STAR Protoc (2021) 2(2):100460. doi: 10.1016/j.xpro.2021.100460 33899027 PMC8055705

[58] Yang C Xia BR Zhang ZC Zhang YJ Lou G Jin WL. Immunotherapy for ovarian cancer: Adjuvant, combination, and neoadjuvant. Front Immunol (2020) 11:577869. doi: 10.3389/fimmu.2020.577869 33123161 PMC7572849

[59] Kuroki L Guntupalli SR. Treatment of epithelial ovarian cancer. BMJ (2020) 371:m3773. doi: 10.1136/bmj.m3773 33168565

[60] Hama N Kobayashi T Han N Kitagawa F Kajihara N Otsuka R. Interleukin-34 limits the therapeutic effects of immune checkpoint blockade. Iscience. (2020) 23(10):101584. doi: 10.1016/j.isci.2020.101584 33205010 PMC7648133

[61] Tokunaga R Zhang W Naseem M Puccini A Berger MD Soni S. CXCL9, CXCL10, CXCL11/CXCR3 axis for immune activation–a target for novel cancer therapy. Cancer Treat Rev (2018) 63:40–7. doi: 10.1016/j.ctrv.2017.11.007 PMC5801162 29207310

[62] Chow MT Ozga AJ Servis RL Frederick DT Lo JA Fisher DE. Intratumoral activity of the CXCR3 chemokine system is required for the efficacy of anti-PD-1 therapy. Immunity. (2019) 50(6):1498–512.e5. doi: 10.1016/j.immuni.2019.04.010 31097342 PMC6527362

[63] Mantovani A Allavena P Marchesi F Garlanda C. Macrophages as tools and targets in cancer therapy. Nat Rev Drug Discovery (2022) 21, 1–22. doi: 10.1038/s41573-022-00520-5 35974096 PMC9380983

[64] El-Gamal MI Al-Ameen SK Al-Koumi DM Hamad MG Jalal NA Oh CH. Recent advances of colony-stimulating factor-1 receptor (CSF-1R) kinase and its inhibitors. J Med Chem (2018) 61(13):5450–66. doi: 10.1021/acs.jmedchem.7b00873 29293000

[65] Xu J Escamilla J Mok S David J Priceman S West B. CSF1R signaling blockade stanches tumor-infiltrating myeloid cells and improves the efficacy of radiotherapy in prostate cancer. Cancer Res (2013) 73(9):2782–94. doi: 10.1158/0008-5472.CAN-12-3981 PMC4097014 23418320

[66] Ries CH Cannarile MA Hoves S Benz J Wartha K Runza V. Targeting tumor-associated macrophages with anti-CSF-1R antibody reveals a strategy for cancer therapy. Cancer Cell (2014) 25(6):846–59. doi: 10.1016/j.ccr.2014.05.016 24898549

[67] Mok S Koya RC Tsui C Xu J Robert L Wu L. Inhibition of CSF-1 receptor improves the antitumor efficacy of adoptive cell transfer ImmunotherapyCSF-1R blockade improves immunotherapy. Cancer Res (2014) 74(1):153–61. doi: 10.1158/0008-5472.CAN-13-1816 PMC3947337 24247719

[68] Kumar V Donthireddy L Marvel D Condamine T Wang F Lavilla-Alonso S. Cancer-associated fibroblasts neutralize the anti-tumor effect of CSF1 receptor blockade by inducing PMN-MDSC infiltration of tumors. Cancer Cell (2017) 32(5):654–68.e5. doi: 10.1016/j.ccell.2017.10.005 29136508 PMC5827952

[69] Magkouta SF Vaitsi PC Pappas AG Iliopoulou M Kosti CN Psarra K. CSF1/CSF1R axis blockade limits mesothelioma and enhances efficiency of anti-PDL1 immunotherapy. Cancers. (2021) 13(11):2546. doi: 10.3390/cancers13112546 34067348 PMC8196870

[70] Holmgaard RB Brachfeld A Gasmi B Jones DR Mattar M Doman T. Timing of CSF-1/CSF-1R signaling blockade is critical to improving responses to CTLA-4 based immunotherapy. Oncoimmunology. (2016) 5(7):e1151595. doi: 10.1080/2162402X.2016.1151595 27622016 PMC5006914

[71] Zhu Y Knolhoff BL Meyer MA Nywening TM West BL Luo J. CSF1/CSF1R blockade reprograms tumor-infiltrating macrophages and improves response to T-cell checkpoint immunotherapy in pancreatic cancer ModelsCSF1R blockade improves checkpoint immunotherapy. Cancer Res (2014) 74(18):5057–69. doi: 10.1158/0008-5472.CAN-13-3723 PMC4182950 25082815

[72] Jia SN Han YB Yang R Yang ZC. Chemokines in colon cancer progression. Semin Cancer Biol (2022) 86(Pt 3):400–7. doi: 10.1016/j.semcancer.2022.02.007 35183412

[73] Britt KL Cuzick J Phillips KA. Key steps for effective breast cancer prevention. Nat Rev Cancer. (2020) 20(8):417–36. doi: 10.1038/s41568-020-0266-x 32528185

[74] Fu C Zhu X Xu P Li Y. Pharmacological inhibition of USP7 promotes antitumor immunity and contributes to colon cancer therapy. OncoTargets Ther (2019) 12:609. doi: 10.2147/OTT.S182806 PMC6339463 30697058

[75] Jure-Kunkel M Masters G Girit E Dito G Lee F Hunt JT. Synergy between chemotherapeutic agents and CTLA-4 blockade in preclinical tumor models. Cancer Immunology Immunother (2013) 62(9):1533–45. doi: 10.1007/s00262-013-1451-5 PMC3755230 23873089

[76] Naimi A Mohammed RN Raji A Chupradit S Yumashev AV Suksatan W. Tumor immunotherapies by immune checkpoint inhibitors (ICIs); the pros and cons. Cell Communication Signaling (2022) 20(1):1–31. doi: 10.1186/s12964-022-00854-y 35392976 PMC8991803

[77] Han N Baghdadi M Ishikawa K Endo H Kobayashi T Wada H. Enhanced IL-34 expression in nivolumab-resistant metastatic melanoma. Inflammation Regeneration. (2018) 38(1):1–5. doi: 10.1186/s41232-018-0060-2 29515691 PMC5836392

[78] Ge Y Huang M Yao Y-m. Immunomodulation of interleukin-34 and its potential significance as a disease biomarker and therapeutic target. Int J Biol Sci (2019) 15(9):1835. doi: 10.7150/ijbs.35070 31523186 PMC6743287

[79] MacDonald KP Palmer JS Cronau S Seppanen E Olver S Raffelt NC. An antibody against the colony-stimulating factor 1 receptor depletes the resident subset of monocytes and tissue-and tumor-associated macrophages but does not inhibit inflammation. Blood J Am Soc Hematology. (2010) 116(19):3955–63. doi: 10.1182/blood-2010-02-266296 20682855

[80] Wang X Zhang J Hu B Qian F. High expression of CSF-1R predicts poor prognosis and CSF-1R(high) tumor-associated macrophages inhibit anti-tumor immunity in colon adenocarcinoma. Front Oncol (2022) 12:850767. doi: 10.3389/fonc.2022.850767 35444953 PMC9014714

[81] Antoine L Nathan D Laure M Briac C Jean-François M Corinne B. Compliance with night-time overcorrection bracing in adolescent idiopathic scoliosis: Result from a cohort follow-up. Med Eng physics. (2020) 77:137–41. doi: 10.1016/j.medengphy.2020.01.003 31992499

[82] Baghdadi M Endo H Takano A Ishikawa K Kameda Y Wada H. High co-expression of IL-34 and m-CSF correlates with tumor progression and poor survival in lung cancers. Sci Rep (2018) 8(1):418. doi: 10.1038/s41598-017-18796-8 29323162 PMC5765132

[83] Wang B Xu W Tan M Xiao Y Yang H Xia TS. Integrative genomic analyses of a novel cytokine, interleukin-34 and its potential role in cancer prediction. Int J Mol Med (2015) 35(1):92–102. doi: 10.3892/ijmm.2014.2001 25395235 PMC4249750

[84] Gridelli C Rossi A Carbone DP Guarize J Karachaliou N Mok T. Non-small-cell lung cancer. Nat Rev Dis primers. (2015) 1(1):1–16. doi: 10.1038/nrdp.2015.9 27188576

[85] Ruiz-Cordero R Devine WP. Targeted therapy and checkpoint immunotherapy in lung cancer. Surg Pathol Clinics. (2020) 13(1):17–33. doi: 10.1016/j.path.2019.11.002 32005431

[86] Denisenko TV Budkevich IN Zhivotovsky B. Cell death-based treatment of lung adenocarcinoma. Cell Death disease. (2018) 9(2):1–14. doi: 10.1038/s41419-017-0063-y 29371589 PMC5833343

[87] Marzagalli M Ebelt ND Manuel ER eds. Unraveling the crosstalk between melanoma and immune cells in the tumor microenvironment. In: Seminars in cancer biology. 59, 236–250. Academic Press: Elsevier. 10.1016/j.semcancer.2019.08.002 31404607

[88] Pasquali S Hadjinicolaou AV Sileni VC Rossi CR Mocellin S. Systemic treatments for metastatic cutaneous melanoma. Cochrane Database Systematic Rev (2018) 2. doi: 10.1002/14651858.CD011123.pub2 PMC6491081 29405038

[89] Safi M Jin C Aldanakh A Feng P Qin H Alradhi M. Immune checkpoint inhibitor (ICI) genes and aging in malignant melanoma patients: a clinicogenomic TCGA study. BMC Cancer. (2022) 22(1):978. doi: 10.1186/s12885-022-09860-2 36100891 PMC9469583

[90] Ribas A Hamid O Daud A Hodi FS Wolchok JD Kefford R. Association of pembrolizumab with tumor response and survival among patients with advanced melanoma. Jama. (2016) 315(15):1600–9. doi: 10.1001/jama.2016.4059 27092830

[91] Ascierto ML Makohon-Moore A Lipson EJ Taube JM McMiller TL Berger AE. Transcriptional mechanisms of resistance to anti–PD-1 TherapyTranscriptional mechanisms of resistance to anti–PD-1. Clin Cancer Res (2017) 23(12):3168–80. doi: 10.1158/1078-0432.CCR-17-0270 PMC5474192 28193624

[92] Zaretsky JM Garcia-Diaz A Shin DS Escuin-Ordinas H Hugo W Hu-Lieskovan S. Mutations associated with acquired resistance to PD-1 blockade in melanoma. New Engl J Med (2016) 375(9):819–29. doi: 10.1056/NEJMoa1604958 PMC5007206 27433843

[93] Shin DS Zaretsky JM Escuin-Ordinas H Garcia-Diaz A Hu-Lieskovan S Kalbasi A. Primary resistance to PD-1 blockade mediated by JAK1/2 MutationsPrimary resistance to PD-1 blockade. Cancer discovery. (2017) 7(2):188–201. doi: 10.1158/2159-8290.CD-16-1223 27903500 PMC5296316

[94] Sucker A Zhao F Real B Heeke C Bielefeld N Maβen S. Genetic evolution of T-cell resistance in the course of melanoma ProgressionGenetic evolution of T-cell resistance in melanoma. Clin Cancer Res (2014) 20(24):6593–604. doi: 10.1158/1078-0432.CCR-14-0567 PMC8728890 25294904

[95] Restifo NP Marincola FM Kawakami Y Taubenberger J Yannelli JR Rosenberg SA. Loss of functional beta2-microglobulin in metastatic melanomas from five patients receiving immunotherapy. JNCI: J Natl Cancer Institute. (1996) 88(2):100–8. doi: 10.1093/jnci/88.2.100 PMC2248456 8537970

[96] Dunn GP Sheehan KC Old LJ Schreiber RD. IFN unresponsiveness in LNCaP cells due to the lack of JAK1 gene expression. Cancer Res (2005) 65(8):3447–53. doi: 10.1158/0008-5472.CAN-04-4316 15833880

[97] Guillonneau C Bézie S Anegon I. Immunoregulatory properties of the cytokine IL-34. Cell Mol Life Sci (2017) 74(14):2569–86. doi: 10.1007/s00018-017-2482-4 PMC11107603 28258292

[98] Baghdadi M Endo H Tanaka Y Wada H Seino K-i. Interleukin 34, from pathogenesis to clinical applications. Cytokine. (2017) 99:139–47. doi: 10.1016/j.cyto.2017.08.020 28886491

[99] Raggi C Correnti M Sica A Andersen JB Cardinale V Alvaro D. Cholangiocarcinoma stem-like subset shapes tumor-initiating niche by educating associated macrophages. J Hepatology. (2017) 66(1):102–15. doi: 10.1016/j.jhep.2016.08.012 PMC5522599 27593106

[100] Rietkötter E Bleckmann A Bayerlová M Menck K Chuang H-N Wenske B. Anti-CSF-1 treatment is effective to prevent carcinoma invasion induced by monocyte-derived cells but scarcely by microglia. Oncotarget. (2015) 6(17):15482. doi: 10.18632/oncotarget.3855 26098772 PMC4558165

[101] Foucher ED Blanchard S Preisser L Garo E Ifrah N Guardiola P. IL-34 induces the differentiation of human monocytes into immunosuppressive macrophages. antagonistic effects GM-CSF IFNγ. PloS One (2013) 8(2):e56045. doi: 10.1371/journal.pone.0056045 PMC3568045 23409120

[102] Uhlen M Zhang C Lee S Sjöstedt E Fagerberg L Bidkhori G. A pathology atlas of the human cancer transcriptome. Science (2017) 357(6352):eaan2507. doi: 10.1126/science.aan2507 28818916

